# Supplementary material for: Patterns of Intron Gain and Loss in Fungi
Source: PLoS Biol. 2004 Nov 30;2(12):e422. doi: 10.1371/journal.pbio.0020422 (PMC532390; doi:10.1371/journal.pbio.0020422)
Supplement: Table S1 — Also available at http://genes.mit.edu/NielsenEtAl/. (4.3 MB ZIP). [file pbio.0020422.st001.zip › NielsenEtAl/html/147.html]

AN6651.1.NCU09903.1.MG02572.1.FG08532.1


```
 CLUSTAL W (1.82) Multiple Sequence Alignments - Introns Inserted


Sequence 1: NCU09903.1	1110 aa
Sequence 2: MG02572.1	1118 aa
Sequence 3: FG08532.1	1103 aa
Sequence 4: AN6651.1	1109 aa
Alignment Length: 1127 aa
Number Identitical Residues: 658 aa
Alignment Score (without introns) 29988


MG02572.1 	MSVPTIVVKGDGP-APSQADVSAILDTVFNAQSSNASIDAAYALCELFLNSPSVGFRGLN
NCU09903.1	MTVPIMVADGAAP-PVSQADVASILDTVFNAKTSQASIDAAYGLCEVLLQS--YGYRGLE
FG08532.1 	MPHPVTPPSGDAPPPPSQETINDLLNTIFTAKTSASSIDACYGLCEILLSS--VGVAGLN
AN6651.1  	--MPTVVAKTPSAVPPAPQEISSLLQTIFNAETSQQSLDASYALANLLIQS--VGPFSFS
          	   *    .  .. . :   :  :*:*:*.*::*  *:**.*.*.::::.*   *  .:.

MG02572.1 	AYG-VLAEVKKAAADKKSGLRRESAQNLLGALFEKFPPRDAPSEYVFLLQDGGILGCALD
NCU09903.1	FYG-VLSELKKAAADKKSGLKREGAQNLLGALFERFPPRQPASESLLTSPEAGLVACALD
FG08532.1 	DYG-VITEIKKAAADKKSGLRRESGQNLLGAVLERFLPRQPISEVVLLLQDSNLVGVALD
AN6651.1  	QYDFILPEIKKAALDKKNGARRESAMLILGALVERFPPAHPLSEVVFLLQDGGVFHLALD
          	 *. ::.*:**** ***.* :**..  :***:.*:* * .. ** ::   :..:.  ***

MG02572.1 	ALADKGAVVRDAAQYGLDALFGHLSPEALVVGLLPALVNYLSKKTGKWQGTVGALKMMQK
NCU09903.1	ALADKGAVVREAAQYGLDALYNNLSPEALVVGLLPALIAYLEKSTGKWQGTIGAYQLIEK
FG08532.1 	ALSDKGAVVRDAAQYGLDATFGILSPEALITGLLPALVEYLSKKTGKWQGTVGAYKLLQK
AN6651.1  	ALADKGAVVRDAAQYAIDALYAALKPEAKVNALLPAISSYLSRGTGKWQGFVGAYCLIEK
          	**:*******:****.:** :  *.*** : .****:  **.: ****** :**  :::*

MG02572.1 	MADKAKLSIGGTKEEALEKDLLREAMGNKLAGLIPLVEAGMHDLKSEVEKQSLLTMKSLT
NCU09903.1	ISNKAQITIGTTKEEAAEQDVLREAMGTKLARLIPIVEGGMLDMKNEVAKQAVKTMTALT
FG08532.1 	MADKAKVSLGGTKEEAIEKDLLRESLGAKLAGLIPIVEGGMHDLKSEVEKQAVATMNSLT
AN6651.1  	MALDAQMGSG-TKEQELEKDVLREAMGKTLKDLIPLVESGMHDLKNDVVKCACKAMNALT
          	:: .*::  * ***:  *:*:***::* .*  ***:**.** *:*.:* * :  :*.:**

MG02572.1 	DLLTNDDVAPRIPLLIETMQKPSAQSLQKAIHALSMTTFVAIVTSPVLALLTPFLERSLN
NCU09903.1	TLLSNDDVAPRIPLLIETMQHPSPATVQKAIHALSMTTFVAIVTAPVLALLTPFLERSLS
FG08532.1 	TLLSNDDVAPRIPLLVDTMQHPSSQTLQKAIHALSQTTFVAIVTSPVLALLTPFLERSLN
AN6651.1  	TLLSNDDVEPRIPLLIKTMEQPSEQTLQKAIHALSQTTFVAIVTSPVLALLTPLLERSLN
          	 **:**** ******:.**::**  ::******** ********:********:*****.

MG02572.1 	TPTTPQEVLRQTVVITENLTKLVHDPIEARTFLPKLTPGVKNVADRAALPEVREIATRAL
NCU09903.1	NPGTPQEVLRQTVVITENLTKLVHDPIEARTFLPKLQPGIKSVVDRASLPEVREIATRAL
FG08532.1 	SPTTAQEVLRQTVVIVENLTKLVHDPIEARTFLPKLIPGVKSVCDRASLPEVREIAERAL
AN6651.1  	APTTPQETLRQTVVVVENLTKLVHDPAEARTFLPKLKPGVQAVKDRASLPEVRELATRAM
          	 * *.**.******:.********** ********* **:: * ***:******:* **:

MG02572.1 	NVMNKAMAV--DKDNVVERTKGEDVGKILDAEIKRANGLAG--DQDFYKSLRSYISDQVA
NCU09903.1	AVMDKAMGS--DQDKVIERTHAEDVGKVLDAEVQKAGGLVG--DPVVYQTVRKYVGEMVA
FG08532.1 	ATMEKAMGD--DKD-VVARTSGEDVAKVLDEQIKANGGLAS--QQELFKQARPYISDMVA
AN6651.1  	DVMEKAMADKDIAAGSVAKVTPDEVLSVLNAKIQENGGLARPDAATLFELGKTYVSEMVR
          	 .*:***. ..     : :.  ::* .:*: :::  .**. ..   .::  : *:.: * 

MG02572.1 	EDVNHRHVTRIAGRISPYLKSLMKSHEA---VAAAVQKYYVEEDQRKYGVPEKEDDGETE
NCU09903.1	EDVNHRHCNRIAARTGPYLSFLVADPVA---VGEAVQKHYVDEDAIKYGVPEKEDDGEIE
FG08532.1 	IDVNYRYVNRISGRIAPYIKTFLKDAEAPQKVADAVQKYYVEEDERKYGVPEKEDDGEVE
AN6651.1  	EDVNCRLLDRIPSCIAPYLRGLLAENKN-DEVAAAVQAHFVAEDERKYGKPVPDDPNEVE
          	 *** *   **..  .**:  :: .    ..*. *** ::* **  *** *  :* .* *

MG02572.1 	IVNADFSLAYGGMLLLSHTNLRLLKGHRYGLCGRNGAGKSTLMRSIANEKLEGFPPQSVL
NCU09903.1	IVNADFSLAYGGMLLLQHTNLRLLKGHRYGLCGRNGAGKSTLMKAIASGKLEGFPSQDVL
FG08532.1 	IVNADFSLAYGGMLLLSHTNLRLLKGHRYGLCGRNGAGKSTLMRSIANGKLEGFPPQDVL
AN6651.1  	IVNANFSLAYGGMLLLSHTNLRLLKGHRYGLCGRNGAGKSTLMRSIANDKLEGFPPPDQV
          	****:***********.**************************::**. ******. . :

MG02572.1 	RTCYVEHNQGEDADISILDFMVKDPTVAKENYTRQEISDVLAEFGFTAGPEGRQSQKVGS
NCU09903.1	RTCYVEHNQGEDADISILDFMVKDPTIASE--GRERISAVLEEFGFTSGPEGRQSQKVGS
FG08532.1 	RTCYVEHNQGEDADISILEFVAKDPEIATQ--GLERISEVLAEFGFTAGPEGRQSERVGS
AN6651.1  	RTCFVEHNQGEDADLTIFEYVKKDPKIAAE--GDEHIRNVLLEFGFTDGPEGRQSQAVGS
          	***:**********::*:::: *** :* :    :.*  ** ***** *******: ***

MG02572.1 	LSGGWKMKLALARAMLQKADVLLLDEPTNHLDVANIAWLENYLKTHPDITSLIVSHDSKF
NCU09903.1	LSGGWKMKLALARAMLQKADVLLLDEPTNHLDVANIKWLEEYLKSHTDITSLIVSHDSGF
FG08532.1 	LSGGWKMKLALARAMLQRADVLLLDEPTNHLDVANIAWLENYLKSHPDITSLIVSHDSGF
AN6651.1  	LSGGWKMKLALARAMLLKADVLLLDEPTNHLDVANVKWLQEYLKKHTEITSLIVSHDSGF
          	**************** :*****************: **::***.*.:********** *

MG02572.1 	LDEVTTDIYHYEPGKKLGHYKGNLSD~FVKIRPEAKSYYTLSASNVQFKFPPPGILSGIK
NCU09903.1	LDNVTTDIYHYEPNKKLGHYKGNLAA~FVNVRPEAKAYYTLSASNVQFKFPPPGILSGVK
FG08532.1 	LDNVTTDIYHYEPNKKLACYKGNLAN~FVQIKPEAKSYYTLSASTVQFKFPPPGILTGVK
AN6651.1  	LDEVCTDIYHYE-QKKLVCYKGNLAD2FVKVKPEAKSYYTLSASNIQFKFPPPGILSGIK
          	**:* *******  ***  *****:  **:::****:*******.:**********:*:*

MG02572.1 	SMTRAVIRMTNVSYTYPGAPKPSLSDVSCQLTLSSRVAIIGANGAGKSTLIKLLTGEVIP
NCU09903.1	SQTRAIIRMTDVSFTYPNAPKPSLSGVTCQLSLSSRVAIIGPNGAGKSTLIKLLTGEVIP
FG08532.1 	SQTRAIIRMSNVSYAYPNAPKPSLSDVSCQLTLSSRVAIIGPNGAGKSTLIKLLTGETIP
AN6651.1  	SNTRSILRMTDCSYTYPGASKPSLTGASLSLTLSSRVAIIGGNGAGKSTFIKMLTGETIP
          	* **:::**:: *::**.*.****:..: .*:********* *******:**:****.**

MG02572.1 	NSGKVEKHPNLRIGYIKQHALEHVEMHLEKTPNQYLQWRYQHGDDREVHMKQTRVLSEED
NCU09903.1	TAGKVEKHPNLRIGYIKQHALEHVEMHLEKTPNQYLQWRYQHGDDREVHMKQTRQLTEAD
FG08532.1 	STGKVEKHPNLRIGYIKQHALEHVEMHLEKTPNQYLQWRYQHGDDREVHMKQTRALSEAD
AN6651.1  	QTGKVEKHPNLRIGYIKQHALEHVEMHLEKTPSQYLQWRYANGDDREVFLKQTRILTEED
          	 :******************************.******* :******.:**** *:* *

MG02572.1 	RQQMEKPVDLGDGRGPRQLESLVGRQKYKKTFQYEV2KWKAMLPKYNSQISRETLIELGF
NCU09903.1	KEQMDKFVDVGDGKGKRQVEALVGRQKYKKTFQYEV2KWRGFLPKHNTMISRETLLELGF
FG08532.1 	RAQMDKWVDLKDGKSARQVESLVGRQKYKKTFQYEV2KWRGLLPKNNTMISRETLSELGF
AN6651.1  	KAQLEKPVDLGDGRGPRRIEALIGRQKWKKSFQYEV2KWVGLLPKHNTMISRETLLELGF
          	: *::* **: **:. *::*:*:****:**:***** ** .:*** *: ****** ****

MG02572.1 	NKLVQEFDDHEASREGLGYRELQPAVISKHFEDLGLDPEIANHNEIGSLSGGQKVKVVIA
NCU09903.1	QKLIQEFDDHESSREGLGYRELQPAVISKHFEDLGLDPEIANHNEIGSLSGGQKVKVVIA
FG08532.1 	DKLVQEFDDHEASREGLGYRELQPKVISKHFEDLGLDPEIANHNEIGSLSGGQKVKVVIA
AN6651.1  	FKMVQEFDDHEASREGLGFRVLEPKTIAKHFEDVGLDPEIANHNEISGLSGGQKVKVVLA
          	 *::*******:******:* *:* .*:*****:************..**********:*

MG02572.1 	GAMWNNPHLLVLDEPTNFLDRDSLGGLAVAIREFKGGVVMISHNDEFVGALSSEQWHVAD
NCU09903.1	GAMWNNPHMLVLDEPTNFLDRDSLGGLAVAIRDFKGGVVMISHNEEFVGALASETWNVVD
FG08532.1 	GAMWNNPHLLVLDEPTNFLDRDSLGGLAVAIRDFKGGVILISHNEEFVGALCSEQWHVND
AN6651.1  	GAMWNNPHLLVLDEPTNFLDRDSLGGLAVAIRDFKGGVVMISHNEEFVGALCPEQIHIAD
          	********:***********************:*****::****:******..*  :: *

MG02572.1 	GRVAHRGHNAIALDRFEDSRPGSGVTSGAATPNGFASSAVSSAMNSAMNSAVNSGAEDNG
NCU09903.1	GRVTHKGQNSAALDRFEDSKPASAVTSGLNTPMGMS--EPSTAVNSGAED--NSLGVTGE
FG08532.1 	GRVALKGNAAISLDRFEDSRPSSGVNSTAASS------VVSSAVNSGIED--------NS
AN6651.1  	GKIVARTNTAISLDRFEDS-ASSTPQPGSTAASSVANSAAASAVNSGAED--------QG
          	*::. : : : :******* ..*   .   :. . :.:  ::*:**. :.          

MG02572.1 	ELKFKAKKKKKKTKKELKEQEVRRRLRHIEWLNSPKGTPHPPDTDDEAE
NCU09903.1	SMTFKAKKKKKMTKKELKEREARRRLRHIEWLNSPKGTPHPKDTDDEDE
FG08532.1 	DMKFRARKKKKMTKKEVKEREVRRRLRHIEWLNSPKGTPHPPDTDDEDN
AN6651.1  	ELKFKARKKKKMTRAQLKEREARRRLRHIEWLNSPKGTPKPPDTDDEAE
          	.:.*:*:**** *: ::**:*.*****************:* ***** :
```
